# Microvascular significance of TGF-β axis activation in COVID-19

**DOI:** 10.3389/fcvm.2022.1054690

**Published:** 2023-01-06

**Authors:** Lauren M. Arguinchona, Caterina Zagona-Prizio, Megan E. Joyce, Edward D. Chan, James P. Maloney

**Affiliations:** ^1^School of Medicine, University of Colorado Anschutz Medical Campus, Aurora, CO, United States; ^2^Division of Pulmonary Sciences and Critical Care Medicine, University of Colorado Anschutz Medical Campus, Aurora, CO, United States; ^3^Rocky Mountain Regional Veterans Affairs Medical Center, Aurora, CO, United States; ^4^National Jewish Health, Denver, CO, United States

**Keywords:** fibrosis-pulmonary-histopathology-diagnosis, SARS-CoV-2, acute respiratory disease syndrome (ARDS), thrombosis, TGF-β1, TSP1/THBS1, COVID-19

## Abstract

As 2023 approaches, the COVID-19 pandemic has killed millions. While vaccines have been a crucial intervention, only a few effective medications exist for prevention and treatment of COVID-19 in breakthrough cases or in unvaccinated or immunocompromised patients. SARS-CoV-2 displays early and unusual features of micro-thrombosis and immune dysregulation that target endothelial beds of the lungs, skin, and other organs. Notably, anticoagulation improves outcomes in some COVID-19 patients. The protein transforming growth factor-beta (TGF-β1) has constitutive roles in maintaining a healthy microvasculature through its roles in regulating inflammation, clotting, and wound healing. However, after infection (including viral infection) TGF-β1 activation may augment coagulation, cause immune dysregulation, and direct a path toward tissue fibrosis. Dysregulation of TGF-β signaling in immune cells and its localization in areas of microvascular injury are now well-described in COVID-19, and such events may contribute to the acute respiratory distress syndrome and skin micro-thrombosis outcomes frequently seen in severe COVID-19. The high concentration of TGF-β in platelets and in other cells within microvascular thrombi, its ability to activate the clotting cascade and dysregulate immune pathways, and its pro-fibrotic properties all contribute to a unique milieu in the COVID-19 microvasculature. This unique environment allows for propagation of microvascular clotting and immune dysregulation. In this review we summarize the physiological functions of TGF-β and detail the evidence for its effects on the microvasculature in COVID-19. In addition, we explore the potential role of existing TGF-β inhibitors for the prevention and treatment of COVID-19 associated microvascular thrombosis and immune dysregulation.

## Introduction

The transforming growth factor-beta (TGF-β) superfamily consists of 33 known mammalian proteins classified into subfamilies that include bone morphogenetic proteins (BMPs), growth differentiation factors (GDFs), activins, inhibins, and three TGF-β isoforms (TGF-β1, TGF-β2, and TGF-β3) (1). TGF-β1 is a ubiquitous pleiotropic cytokine expressed by numerous cell types and is well-known for its regulation of inflammation and wound healing. TGF-β1 is the dominant protein expressed in most tissues (including immune cells) whereas expression of TGF-β2 and TGF-β3 are more restricted. In this review “TGF-β” will refer to TGF-β1 unless otherwise specified. The amino acid sequence of TGF-β1 is markedly conserved between species, with 100% sequence homology between human, simian, bovine, porcine, murine, and chicken proteins. TGF-β1 shares moderate sequence homology with TGF-β2 and TGF-β3, with some overlapping functions. The phenotypes of null mice for each of these isoforms are strikingly different, suggesting key functions are distinct for each isoform. TGF-β1 null mice have the most severe phenotype, dying at 2 weeks of life from widespread inflammation, while TGF-β2 and TGF-β3 null mice each succumb to developmental abnormalities soon after birth (2–4).

**FIGURE 1:**
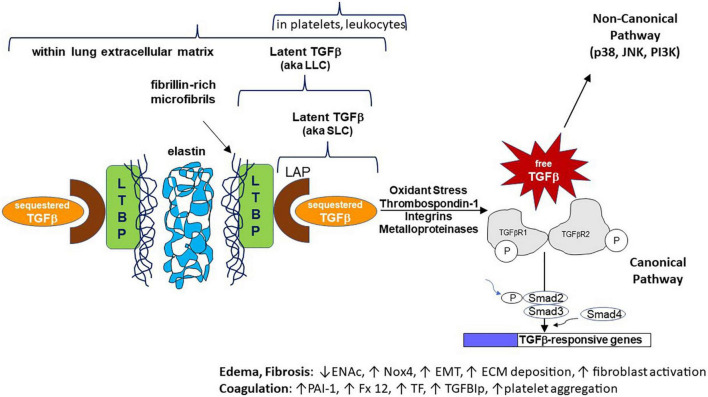
Cartoon schematic representing normal human transforming growth factor-beta (TGF-β) expression, latency, activation mechanisms, intracellular signaling, and effects on specific genes/pathways. The non-canonical signaling pathway is not discussed in this manuscript. For space purposes the binding of active TGF-β as a dimer to its tetrameric receptor complexes is not shown. ENaC, epithelial sodium channel; Nox4, NADPH oxidase 4; EMT, epithelial to mesenchymal transition; ECM, extracellular matrix; PAI-1, plasminogen activator inhibitor 1; Fx 12, coagulation factor 12; TF, tissue factor; TGFBIp, TGF-beta induced protein.

In its most common form, TGF-β is inactive, or “latent” (L-TGF-β), being covalently bound to the latency-associated peptide (LAP) derived from the 5’ end of its gene transcript, which shields its receptor binding sites. This forms the small latent complex (SLC). TGF-β is secreted by most cells in this SLC form. LAP can also form a disulfide bond with the latent TGF-β binding protein (LTBP, from different genes for each TGF-β isoform) forming the large latent complex (LLC) (Figure 1). Interestingly, mice null for LTBP also suffer from widespread inflammation (5). Latency of TGF-β is essential to sequester its pluripotent effects while also maintaining a state where it can be rapidly activated (such as during tissue injury and acute inflammation). These inactive complexes can be manipulated through a variety of pathways that lead to release of active TGF-β, including non-covalent interactions with thrombospondin-1 (TSP1) or integrins, or proteolytic degradation of the LAP by enzymes such as metalloproteinases (5–9). TSP1 is able to conformationally alter LAP through its “KRFK” motif (lysine arginine phenylalanine lysine), one of the few molecular known to activate the latent form of TGF-β (10). Other such “keys” are contained in motifs of ανβ6 and ανβ8 integrins, which typically require membrane or matrix anchoring to bind to RGD amino acid motifs (arginine glycine aspartic acid) in LAP and deform its shape (11). This post-translational regulation of all TGF-β isoforms is the main determinant of their availability, rather than from changes in gene expression. The lung is one organ with high latent TGF-β expression and protein levels, both within structural and immune cells and intercalated into the extracellular matrix (after cell secretion) (Figure 1; 12). Thus in most tissues but particularly in the lungs, inflammatory and other stimuli can rapidly release active TGF-β from its latent stores (13). TGF-β release in this scenario is context-dependent, but often is a key event leading to a post-inflammatory tissue healing phase of illness (1). In some contexts TGF-β has anti-inflammatory effects, whereas in other contexts persistent stimulation by TGF-β may lead to tissue fibrosis (14).

### TGF-β is a mediator of thrombosis

The initial physiological response in mammals to vascular injury is hemostasis which begins with platelet aggregation at the wound site. Platelets are the most abundant source of human TGF-β1 (40–100 times the level of TGF-β1 compared with other cells), and this protein is stored in its latent form in platelet alpha granules (15). Coagulation factors V, XI, and XIII, and the TGF-β activator protein TSP1 are also stored in the alpha granules (15, 16). Therefore, measuring TGF-β levels in blood samples is complex due to much higher TGF-β levels in serum (coagulated blood) compared to that in plasma (anticoagulated blood) (17). Thus, comparison of serum TGF-β levels between populations or during illnesses may be confounded by platelet counts, intrinsic platelet aggregation properties, and/or use of anti-platelet agents (17, 18). Both latent forms of TGF-β are released from platelets with the LLC and SLC conformations comprising 65 and 35%, respectively (19, 20). These latent TGF-β forms are then likely activated by co-secreted platelet enzymes (like furin), co-secreted proteins (TSP1), shear stress (which can double in a thrombosing vasculature), or inflammation-induced enzymes like metalloproteinases (20, 21). Altered release of TGF-β from platelets has been described in several inflammatory diseases (17, 22). Notably, some cancers appear to “hijack” platelets and use TGF-β and other proteins to promote tumor growth and provide a permissive tumor environment (23).

Platelets express TGF-β receptors, so they can respond to TGF-β in an autocrine or paracrine manner (24). In normal platelets, exogenous TGF-β1 induces platelet aggregation (25). Exogenous TGF-β1 may also induce a procoagulant state *via* release of vascular or platelet stores of Factor XII, tissue factor, and plasminogen activator inhibitor-I (PAI-I) (26–28). In SARS-CoV-2 infected lung epithelial cells, the PAI-I gene (SERPINE1) is strongly upregulated (29). TGF-β also indirectly augments platelet activation *via* induction of TGF-β induced protein (TGFBIp) in platelets, respiratory epithelial cells, endothelial cells, keratinocytes, fibroblasts, and monocytes. TGFBIp is secreted and is also stored in the extracellular matrix, accumulating at sites of inflammation. TGFBIp has RGD sequences and fasciclin 1 domains that appear to bind to platelet surface integrins and activate platelets. Transgenic mice that overexpress TGFBIp are more prone to pulmonary embolism (30). Interestingly, platelets also maintain a small amount of L-TGF-β on their external surface bound to the glycoprotein-A repetitions predominant (GARP) protein. However, GARP-bound TGF-β seems more important for immune interactions rather than for coagulation (31).

Transgenic mouse models have given substantial insight into the role of platelet-derived TGF-β1 in coagulation. TSP1 null mice lose the major non-integrin mechanism by which L-TGF-β is activated. These mice have less active TGF-β in both platelets and in healing wounds, defects in platelet aggregation and wound healing, and markedly less platelet TGF-β release during aggregation (32). TGF-β1 null mice are difficult to study due to their demise *in utero* or shortly after birth, but conditional knockout of TGF-β1 in megakaryocytes (platelet precursors) reveals lower blood TGF-β1 levels and a bleeding diathesis with decreased platelet aggregation from fibrinogen receptor binding defects (25). In summation, existing evidence places TGF-β as an important orchestrator of coagulation whose role may loom larger during wound healing following inflammation. The key studies supporting this conclusion are summarized in Table 1.

**TABLE 1:** Key evidence for transforming growth factor-beta (TGF-β) as a mediator of thrombosis.

References	Key findings
Assoian et al. (15)	Identification of TGFβ; description of high expression in human platelets
Huber et al. (23)	Human platelet TGFβ is principally in the latent form and is resistant to protease digestion
Grainger et al. (19)	Human platelet TGFβ exists as both the LLC and SLC forms; plasmin releases additional TGFβ from clots
Crawford et al. (16)	TGFβ in platelets is bound to thrombospondin-1 (an key activator of latent TGFβ)
Hoying et al. (25)	TGFβ enhances platelet aggregation *via* effects on the fibrinogen receptor
Dong et al. (28)	TGFβ activity correlates with PAI-I expression in vascular endothelium; TGFβ increases PAI-I expression in vascular cells
Lev et al. (24)	Platelets have TGFβ receptors; TGFβ1 does not cause direct platelet aggregation but modulates ADP-induced aggregation
Ahamed et al. (21)	Shear stress causes activation of circulating latent TGFβ (present in the LLC form), *in vivo* clotting releases TGFβ
Kim et al. (30)	TGFβ indirectly activates platelets through platelet TGFβ induced protein, which potently increases *in vivo* clotting
Jablonska et al. (26)	TGFβ induces coagulation factor XII *via* a SMAD binding element in its gene promoter
Meyer et al. (18)	Targeted genetic ablation of platelet TGFβ decreases cardiac fibrosis after injury and decreases circulating TGFβ
Maloney et al. (17)	Platelet TGFβ release is decreased in chronic inflammatory lung disease (cystic fibrosis)
Saito et al. (27)	Platelet TGFβ induces tissue factor expression

All listed studies are cited in the references section. TGF-β, transforming growth factor beta; LLC, large latent complex; PAI-I, plasminogen activator inhibitor; SLC, small latent complex; SMAD, small mothers against decapentaplegic (transcription factor named as an anagram after homologies with fly and worm genes).

### TGF-β has critical roles in inflammation and immune regulation

Transforming growth factor-beta (TGF-β1) is the major TGF-β isoform involved in the regulation of immunity (12). Augmented TGF-β1 signaling drives a local suppression of the host immune system *via* direct inhibition of effector immune cells such as T cells, natural killer (NK) cells, and dendritic cells. This effect is amplified by activation of immune suppressor T cells known as T regulatory cells (Tregs) (33). Tregs further suppress T effector cell, dendritic cell, and NK cell function–thus limiting their responses to invading pathogens and blunting inflammation (34–36). Much of this occurs due to direct effects of SMAD transcription factors. TGF-β1 binding to its receptors leads to intracellular phosphorylation of SMAD3 and SMAD4 transcription factors, which then enter the nucleus and affect expression of multiple genes that leads to an inhibition of inflammatory responses (37). Notably, stimulated Tregs also secrete TGF-β1 with paracrine effects on neighboring cells, and keep a small amount of L-TGF-β1 on their surface bound to the GARP protein (31). On balance, these TGF-β1 driven pathways dampen inflammation on route to wound healing. The importance of these predominantly anti-inflammatory pathways is illustrated by the inflammatory phenotype of the TGF-β1 null mouse, which dies several weeks after birth from widespread inflammation in multiple organs (2).

However, TGF-β1 is pleiotropic and can also stimulate immune responses and drive inflammation through the Th17 pathway, particularly in the context of high IL-6 activity (38). TGF-β1 and IL-6 cooperatively drive induction of Th17 cells and secretion of proinflammatory IL-17 (39). Th17 pathway activation may also contribute to tissue fibrosis (40). Furthermore, IL-17 is a potent neutrophil chemokine and increased absolute neutrophil number, percentage of neutrophils, and neutrophil: lymphocyte ratio in the blood of COVID-19 patients are predictive of progression to severe disease (41).

### TGF-β dysregulation in ARDS and pulmonary fibrosis

Prior to the COVID-19 pandemic, there was strong evidence that TGF-β1 is a key driver in the pathophysiology of the acute respiratory distress syndrome (ARDS) and in chronic fibrotic lung diseases such as idiopathic pulmonary fibrosis (IPF). In the early phases of ARDS, inflammatory signals likely release active TGF-β1 from latent stores in the lung extracellular. Released TGF-β1 can immediately inhibit alveolar fluid reabsorption through rapid endocytosis of the apical sodium channel (ENAc) at the airway surface of alveolar type II cells and drive oxidant stress through NADPH oxidase 4 (NOX4) induction (42–44). TGF-β1 also downregulates prolyl hydroxylase 2 (PHD2), and thereby stabilizes the transcription factor hypoxia inducible factor alpha (HIF-1α) leading to further upregulation of hypoxia-inducible genes such as mediators of endothelial permeability (45). TGF-β1 null mice die of multi-organ inflammation, including lung inflammation with features of ARDS. Thus, the complete absence of TGF-β1 and its related immune suppressive functions also favors lung inflammation, suggesting that TGF-β1 dysregulation in either direction has effects on inflammation. Interestingly, TSP1 null mice display milder chronic lung inflammation as their principal phenotype and can be rescued to a near-normal state by administration of short peptides of the L-TGF-β1 activating motif from TSP1, suggesting that TGF-β activation may be the most important constitutive function of TSP1 (16).

Transforming growth factor-beta (TGF-β1) staining is prominent in human ARDS lung, and TGF-β1 concentrations are elevated in ARDS bronchoalveolar lavage (BAL) specimens, but are minimal in BAL of healthy controls (42, 46–49). Continued TGF-β signaling akin to what occurs in IPF may drive a small subgroup of patients to develop fibroproliferative ARDS and permanent fibrosis. To date, no TGF-β1 targeted clinical trials have been published in non-COVID ARDS.

Transforming growth factor-beta (TGF-β1) has a clear pathologic role in the chronic disease pathophysiology of IPF. TGF-β1 staining is prominent in the IPF lung, and active TGF-β1 concentrations are elevated in IPF BAL specimens, far exceeding TGF-β2 and TGF-β3 levels (whereas free TGF-β3 levels are highest in healthy lung BAL) (50). Transient adenoviral over-expression of TGF-β1 in mouse lungs causes progressive pulmonary fibrosis that becomes severe by day 60, whereas TGF-β3 overexpression displays mild histologic fibrosis that mostly resolves by day 60 (51). Of the TGF-1β isoforms, TGF-β1 has the most robust effects on differentiation of fibroblasts into activated myofibroblasts by epithelial to mesenchymal transition, upregulating TGF-β receptors, increasing connective tissue growth factor, driving collagen synthesis, and inhibiting degradation of collagenous intercellular matrix (1, 51). To date, two clinical trials of anti-TGF-β drugs in IPF have failed to show a significant clinical benefit (an anti-TGF-β antibody, and an oral integrin blocker; unpublished), while one oral agent (epigallocatechin-3-gallate) showed benefit in a single center study (52).

### Viral evasion of host immune recognition is aided by “TGF-β hijacking”

The principal fitness advantage of SARS-CoV-2 occurs through gain-of-function mutations of its spike protein that enhance infectivity *via* stronger binding to the angiotensin converting enzyme 2 (ACE2) cell entry receptor. While the main immune alteration in response to severe SARS-CoV-2 infection is the tendency toward a pro-inflammatory state, in part triggered by the spike protein, it is less clear how SARS-CoV-2 can escape immune detection during early infection. This leads to its unique asymptomatic carriage in many hosts, only to cause inflammatory illness much later post-exposure than other respiratory viruses.

Curiously, mechanisms to increase TGF-β tissue levels have been exploited by microbes for eons as a path to evade host immune detection, to increase infectivity, and to achieve latency/chronic infection. All of these paths augment a microbe’s reproductive fitness. Even an animal as primitive as a sea anemone employs TSP1 motifs to regulate the activation of L-TGF-β that permits its primitive immune system to tolerate symbiotic algae colonization. Mutation of these motifs leads to rejection of algae and death of the anemone (35, 53). L-TGF-β1 is highly expressed in airway and alveolar epithelium, alveolar macrophages, and in the lung intercellular matrix where inhaled microbes encounter host defenses during their pulmonary phase (12). Augmented TGF-β1 signaling initially drives a local suppression of the host immune system *via* direct inhibition of effector immune cells like T cells and dendritic cells, an effect amplified by activation of immune suppressor Tregs. Tregs further suppress the function of T effector cells, dendritic cells, and NK cells–thus limiting their responses to the invading pathogen (34–36). These effects typically occur due to direct binding of SMAD transcription factors, activated by TGF-β receptors, to gene promoters of IL-2, IL-10, forkhead box P3 protein (FOXP3) and other key immune cytokines and transcription factors (37). TGF-β1 also drives class switching of plasma cells from IgM and IgG to IgA antibody formation even outside mucosal barriers. Since IgA does not fix complement, it is less effective at directing immune responses to an invading pathogen (54).

Augmentation and exploitation of host TGF-β activity (known as “TGF-β hijacking”) by microbes occurs by various mechanisms. Since no known pathogen synthesizes TGF-β, host TGF-β must be hijacked from the host. The hepatitis C virus increases host TGF-β and TSP1 gene expression, increasing activation of latent TGF-β (55). *Trypanosoma cruzi* also increases host TSP1 (56). The influenza virus produces a protease that degrades LAP and thereby releases active TGF-β (57). The SARS 2004 (aka SARS-CoV) virus nucleocapsid protein associates with the TGF-β intracellular signaling transcription factors SMAD2 and SMAD3 and augments TGF-β induced gene transcription (58). Malaria and cryptosporidium parasites produce a TSP-like protein that activates latent TGF-β through TSP1-like WXXW domains (tryptophan, any amino acid, any amino acid, tryptophan) that inhibit the LAP-TGF-β interaction (59, 60). Schistosoma induces host monocyte TSP1 to activate pulmonary TGF-β as a contributor to the pulmonary vascular disease typical with this chronic infection (61, 62). Mitigating the TGF-β exploitation by these microbes consistently leads to decreased infectivity. For example, transgenic mice specifically lacking bronchial epithelial cell TGF-β1 display robust protection from influenza-induced weight loss, airway inflammation, and tissue injury; these airway null mice also have a heightened antiviral immune response (63). There is emerging evidence that SARS-CoV-2 has also evolved to exploit TGF-β and increase its infectivity. Parallel immunoprecipitation studies of SARS-CoV-2 or SARS-CoV-infected human respiratory epithelial cells (A549) reveal a strong and exclusive interaction of the open reading frame 8 (ORF8) protein of SARS-CoV-2 with TGF-β1, TGF-β2, TGFBR2, LTBP1 (and thus latent TGF-β1) that was not seen with SARS-CoV infection. The mechanism for these interactions is unclear, as the small ORF8 protein has no known amino acid sequences for activating LAP, LTBP, or for TGF-β isoform binding such as KRFK, RGD, or WXXW motifs. Whether the ORF8 interaction leads to TGF-β1 activation is unknown (29). Moreover, the TGF-β induced genes fibronectin (FN1) and plasminogen activator inhibitor (PAI-I, SERPINE1 gene) were prominently upregulated, but this was not seen with SARS-CoV infected cells. Another study found that phosphorylation and activation of the TGF-β pathway is prominent in SARS-CoV-2 infection (64). These findings suggest that exploitation of host TGF-β is a key mechanism of SARS-CoV-2 infection, and that inhibition of this pathway is a promising treatment for SARS-CoV-2 infection.

Well-characterized immune responses that occur during SARS-CoV-2 infection include aberrant activity of both the innate and adaptive immune systems (65). Cell-mediated innate immunity functions by activating toll like receptors (TLRs) and triggering interferon (IFN) production at sites of infection (66). Cytokines released by pro-inflammatory macrophages also play a key role in antiviral defense, activating T cells which then produce IFN-gamma and cytokines that activate more macrophages, driving a loop of anti-viral inflammation (67, 68). One hypothesis is that interference with IFN production is a key perturbation that allows SARS-CoV-2 to replicate freely during early infection. In later stages of severe disease, a rebound IFN response can be exaggerated–leading to cytokine storm and inflammation. Complement is also activated in SARS-CoV-2 and animal studies have shown that inhibition of complement may be beneficial during infection (69). The formation of neutrophil extracellular traps (NETs) is critical to the disruption of the microvasculature, with an imbalance in formation and degradation driving inflammation and occlusive NET-derived immunothrombosis (70). Adaptive immunity also plays a key role, with B cells producing multiple classes of antibodies. The production of these antibodies does not occur until at least day 7–8 of infection, and delays in antibody production have been associated with more severe COVID-19 illness (54). T cells are also activated with a predominance of CD4+ and CD8+ cells. Novel T cell responses are seen in SARS-CoV-2 infection, with increased T cell death by apoptosis and increased syncytia activity, presumably induced by SARS-CoV-2, which when internalized can kill T cells. It has also been observed that highly active CD16+ T cells are linked to microvascular endothelial cell injury and these cells can persist after acute infection which may contribute to long COVID. Other key players include pro-fibrotic CD163-expressing monocyte derived macrophages, and NK cells that exhibit impaired anti-fibrotic function (65). This review will focus on the multi-faceted TGF-β response seen with SARS-CoV-2 infection.

### Linking TGF-β activation with thrombo-inflammatory responses in COVID-19

SARS-CoV was a much less prevalent coronavirus that also demonstrated TGF-β dysregulation, with frequent ARDS and pulmonary fibrosis, but it caused less than a thousand deaths (71). Using human peripheral lung epithelial (HPL) cell models infected with SARS-CoV, Zhao et al. found high levels of TGF-β expression, leading to increased plasminogen activator inhibitor-1 (PAI-1) related tissue fibrosis (58). This mechanism of TGF-β induced expression of PAI-1 has been well-described in prior models of lung fibrosis (72). In this same model of HPL cells, low levels of SMAD3/4 TGF-β induced apoptosis were found, leading to persistence of virus infected cells. The SARS-CoV papain-like protease was also shown to induce TGF-β signaling including fibrotic pathways in lung epithelial cells and blood monocytes (73, 74). Elevated levels of TGF-β1 in blood and high expression in autopsied lung were also reported in SARS 2003 patients (75, 76). Therefore, it is thought that TGF-β played an important role in the pathogenesis of SARS-CoV by promoting replication and proliferation of the virus and contributing to coagulation dysregulation and lung fibrosis.

SARS-CoV-2 infection has also been associated with dysregulation of the TGF-β pathway, resulting in increased TGF-β levels and pathway activity. Signatures of increased TGF-β activity in COVID-19 patients include increased plasma and serum TGF-β1 levels (77, 78), increased RNA levels of TGF β-induced protein in the blood of ARDS patients (79), plasmablast and monocyte TGF-β signatures in blood (80), increased IgA class switching pathognomonic of a TGF-β effect (81), and TGF-β mediated suppression of circulating NK cells (78). Furthermore, increased blood TGF-β levels were characteristically correlated with increased disease severity (80, 82). Strikingly, myeloid-derived suppressor cells (MDSC) from COVID-19 patients suppress T effector cells but this effect can be blocked with a neutralizing antibody to TGF-β, and this TGF-β antibody also rescued interferon responses in MDSC to SARS-CoV-2 antigens, where the number of MDSC also correlated with plasma TGF-β1 levels (83).

Another function of TGF-β is to dampen excessive immune responses and maintain homeostasis. This effect is unfortunately aberrant in SARS-CoV-2 infection due to the timing of its expression. In the sera of patients with severe COVID-19 infections, TGF-β gene expression was found to peak during the first 2 weeks of infection (78). At this time, NK cells, a key part of the innate immune system with activity against RNA viruses, were found to be inhibited in a TGF-β-dependent fashion. After pretreatment with TGF-β blocking antibodies, NK cell function recovered (78). This shows that it is not only the expression, but also the timing of TGF-β responses that allows the virus to proliferate, undetected and undisturbed by the immune system. In parallel, TGF-β1 could also contribute to inflammation through Th17 pathway activation in an IL-6 rich environment such as in the pulmonary phase of COVID-19 (67, 84–86). Interestingly, a subset of SARS-CoV-2 infections leads to a multisystem inflammatory syndrome in children (MIS-C; can also occur in adults) similar to Kawasaki’s disease–a disease associated with common genetic variation in the TGF-β pathway (87, 88).

#### Evidence of TGF-β as a mediator of lung microvascular injury and thrombosis during COVID-19

Most COVID-19 deaths occur from ARDS. While ARDS was also the major cause of death in SARS 2004, our understanding of lung pathophysiology in the SARS 2004 pandemic was limited by its brief duration and limited mortality (89). Due to its longer pandemic duration and an extensive number of investigations, substantial insights into COVID-19 ARDS have been made. Many of these insights resulted from its comparison to data from investigations of ARDS due to influenza, bacterial, and other etiologies (designated here as non-COVID ARDS). Compared to non-COVID ARDS, COVID-19 ARDS has evidence of more extensive microvascular thrombosis (in skin and lung) and a higher propensity to cause lung fibrosis (90, 91).

Microvascular clotting and venous thromboembolism are more common in COVID ARDS (90). Empiric treatment dose anticoagulation has been shown to benefit hospitalized COVID-19 patients outside of intensive care units (ICU), but not in severely-ill patients–suggesting that full dose anticoagulation may come too late in these patients to be of benefit (this subgroup still benefits from prophylactic anticoagulation) (92). The severity of microvascular clotting in COVID-19 ARDS has been surprising, with extensive clotting in lung and skin far surpassing that seen in non-COVID ARDS, with no clear explanations (93, 94).

In one study, postmortem lung samples and antemortem plasma samples were collected from a cohort of COVID-19 ARDS patients. In these samples, TGF-β expression and blood TGF-β1 levels paralleled PAI-1 expression in fibrotic areas of lung and in the endothelium of alveolar septal capillaries and blood vessels (95). PAI-1, a potent inhibitor of fibrinolysis, would be expected to promote clotting in these regions (28). This correlation of TGF-β with PAI-1 highlights an imbalance between prothrombotic and antifibrinolytic pathways in COVID-19. In autopsy studies of patients who died from COVID-19 versus those who died of causes without ARDS, there was a significant increase in expression of TGF-β1 in COVID-19 lungs (96). This TGF-β increase was associated with activation of the Th2 pathway, since increased immune expression of sphingosine-1 (M2 macrophages) and IL-4 were found. M2 macrophages are known to stimulate TGF-β which, in turn, potentiates tissue fibrosis and ineffective pathways for viral clearance. Most COVID-19 autopsy studies have not reported TGF-β staining.

Elevated blood TGF-β1 levels are described in multiple reports of COVID-19 cohorts and may also be a biomarker of COVID ARDS severity. Colarusso et al. described higher plasma TGF-β1 levels and higher stimulated release of TGF-β1 from blood monocytes in patients with signs of lung fibrosis on imaging (97, 98). Ghazavi et al. found increased serum TGF-β1 levels in COVID-19 patients that correlated positively with disease severity and serum IL-17 (82). Serum TGF-β1 levels have also been show to correlate with circulating monocyte phenotype in severe COVID-19 (99). In another study, blood levels of TGF-β-induced protein (TGF-βIp) were markedly elevated in severe COVID-19 (79). This protein is of particular interest as its transgenic overexpression worsens pulmonary emboli in a murine model of venous thromboembolism (30). The cause of increased blood TGF-β1 and activity in COVID-19 is likely in large part related to elevated pro-inflammatory cytokines and oxidant stress. IL-1 and IL-17 are proinflammatory cytokines that are typically elevated in blood of severe COVID-19 patients. IL-6 increases TGF-β1 expression, and IL-17 increases TGF-β receptor II expression (100, 101). TGF-β-induced epithelial-to-mesenchymal transition (EMT) *via* upregulation of NOX4 expression and activity that is augmented by intracellular oxidant stress (102). Thus, inhibition of TGF-β production by IL-6 may be one mechanism underlying the survival benefit seen with IL-6 inhibitor administration during severe COVID-19 (84). Likewise, corticosteroids improve survival in hypoxic COVID-19 patients, and steroid effects include suppression of TGF-β1 gene expression (103, 104).

Assessment of airspace levels of TGF-β or airway immune cell TGF-β expression during COVID-19 ARDS have been limited to a few reports based on understandable desire to limit aerosolization of sputum from COVID-19 patients to research or clinical personnel. While multiple groups have reported on RNA expression or inflammatory cell distributions within BAL fluid from mechanically ventilated COVID-19 patients, to our knowledge none have reported an assessment of TGF-β protein levels in BAL fluid or other respiratory specimens. Grant et al. collected BAL from 88 COVID-19 patients and reported RNA sequencing results on 10 specimens and cell subset analyses by flow cytometry; in this study TGF-β was not reported as a dysregulated gene nor was a TGF-β driven gene signature reported among findings of T cell and macrophage drive alveolar inflammation (68). Ronit et al. studied lymphocyte subsets and a limited cytokine panel from BAL of four COVID-19 patients and found an inflammatory pattern, but TGF-β was not a target of the assays (105). Xiong et al. reported that RNA sequencing results from two COVID-19 BAL specimens demonstrated significantly increased TGF-β2 gene expression in BAL cells compared to three healthy controls (106). Zhou et al. reported RNA sequencing results from cells of eight COVID-19 BAL specimens compared to healthy control and non-COVID pneumonia patients, but TGF-β genes were not reported as dysregulated. Ferreira-Gomes et al. reported day 31 and day 46 BAL cell analysis from one mechanically-ventilated COVID-19 ARDS patient and found persistently high TGF-β protein in T cells (80). Since TGF-β availability is regulated at the post-transcriptional level, measurement of active TGF-β levels in lung secretions and lung tissue will be important to assess.

Cell-based models of respiratory epithelial cell infection with SARS-CoV-2 have provided additional insights into the role of TGF-β in epithelial cell injury. In an interactome study, human A549 lung epithelial cells transfected with individual SARS-CoV-2 proteins showed a prominent and exclusive binding of the viral ORF8 protein with TGF-β1-2 isoforms, TGF-β receptor 2 (TGFβR2), and L-TGF-β (29). This finding was unexpected, as the small ORF8 protein has no known protein sequences for LAP, LTBP, or TGF-β isoform binding such as KRFK, RGD, or WXXW motifs (107, 108). Moreover, known TGF-β1 induced genes fibronectin and PAI-1 were upregulated.

A recent animal model of COVID-19 used the MA10 mouse strain to evaluate longitudinal changes in lung pathology with a non-lethal SARS-CoV-2 dosing approach. This model revealed a post-transcriptional pattern of increased alveolar TGF-β1 pathway signaling (such as fibronectin and type I collagen upregulation) at days 15 and 30 post-infection. Moreover, increased TGF-β1 gene transcription was seen in areas of residual fibrosis at 15–120 days after clearance of viral infection (109).

#### Evidence of TGF-β as a mediator of skin microvascular injury and thrombosis during COVID-19

COVID-19 is associated with variable dermatologic presentations with vasculopathies being common. The rich capillary supply network of skin and its safe and feasible biopsy have given important insights into the microthrombotic and inflammatory features of COVID-19. Of particular interest is the ability of skin biopsies to give information on living COVID-19 patients. In comparison, study of tissues such as lung or heart microvascular injury during COVID-19 are mostly limited to animal or human autopsy studies.

Prevalence estimates for COVID-19 skin disease are confounded by the predominance of case reports, nevertheless, data from reviews suggest that dermatologic microvascular damage may be present in up to 20–25% of cases, and in almost all severe cases (110). Of these, the most frequently reported conditions are chilblain-like vasculitic acral lesions (pernio-like lesions, “COVID toe”) and reticular lesions such as livedo reticularis and retiform purpura, typically associated with microvascular clotting (110). Several pathophysiologic mechanisms have been implicated in the development of these cutaneous microvascular lesions. In one study, microthrombosis was seen in 87% of premortem skin biopsies of severe COVID-19 cases with ARDS, but no microthrombi were seen in the premortem skin biopsies of mild COVID-19 cases or in skin from ARDS patients with non-COVID etiologies (111). As to inflammatory mediators in COVID-19 affected skin, terminal complement C5b-9 and MASP2 (lectin complement pathway) deposition are prominent in skin biopsies of severely ill COVID-19 patients with livedoid or purpuric cutaneous reactions that clinically suggest vasculitis. Strikingly, deposition of C5b-9 has also been demonstrated in biopsies of apparently normal skin–where its deposition tracks COVID-19 severity (highest in COVID-19 ARDS), while such C5b-9 deposition is absent from the skin of patients with non-COVID ARDS (69, 111). This complement deposition damages the endothelium resulting in further clotting cascade activation and a pro-coagulative state, likely explaining the tight correlation of complement deposition with micro-thrombosis. Complement activation has also been demonstrated in acro-ischemic chilblain-like lesions frequently seen in milder COVID-19 cases and in younger patients (112).

Prominent expression of the transcription factor SIN3A has been demonstrated in premortem skin biopsies of severe COVID-19 patients, where it also correlates with disease severity (111). The potential significance of this finding in the context of TGF-β is that SIN3A is known to co-localize with SMAD transcription factors on TGF-β driven gene promoters, suggesting it may have a cooperative effect with TGF-β on the genesis of microvascular injury during COVID-19 (113). To our knowledge, no biopsy studies have directly evaluated levels of skin TGF-β protein or RNA expression in COVID-19. At autopsy, skin is among the highest ACE2 receptor expressing organs in COVID-19 patients (second only to the lungs) (69). Binding of spike protein (“pseudovirus”) to dermal microvessel ACE2 also correlates strongly as a signal for subsequent complement deposition and inflammation (110, 114). These reports demonstrate unique pathophysiologic features linked with cutaneous microvascular damage, overall disease severity, and patient outcomes. This evidence suggests an interplay of several inflammatory pathways in the pathogenesis of microvascular skin conditions in COVID-19. Further high-quality clinical studies are needed to fully elucidate the pathophysiology of these microvascular manifestations as well as their clinical significance in relation to overall COVID-19 severity and treatment options. Importantly, skin biopsies in living subjects remain a feasible and minimally invasive technique to better understand the longitudinal microvascular pathophysiology of COVID-19, particularly related to investigation of TGF-β pathways. Importantly, anti-TGF-β therapies have been shown to have dramatic effects on TGF-β driven genes in analyses of skin biopsy from clinical trial subjects with scleroderma (115). Analysis of skin biopsies from COVID-19 patients treated with anti-TGF-β therapies also promise to be a useful, safe, and feasible window in understanding the effects of these therapies on microvascular injury due to inflammation and clotting.

**FIGURE 2:**
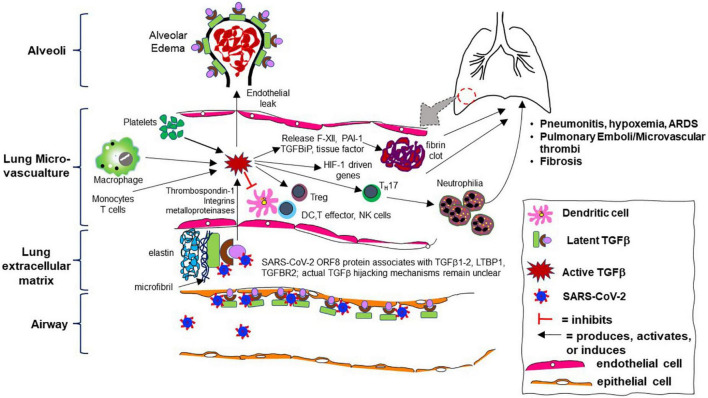
Cartoon schematic of aberrant transforming growth factor-beta (TGF-β) pathway activity during SARS-CoV-2 infection in the human lung microvasculature and its consequences. Skin, kidney, and other organ involvement are not depicted but similar mechanisms likely occur.

These data show that TGF-β is a key mediator in the pathogenicity of COVID-19 and likely vital to the virus’s asymptomatic carriage that contributes to high viral loads and increased viral spread, delayed progression to severe illness, and transition in a subset of patients into a pro-inflammatory, pro-fibrotic final phase of illness with prominent microvascular clotting. A summary of the key evidence for this paradigm during COVID-19 illness is listed in Table 2 and illustrated by Figure 2.

**TABLE 2:** Key evidence for transforming growth factor-beta (TGF-β) as a mediator of COVID-19 immune dysregulation and thrombosis.

References	Key findings
Park et al. (79)	TGFβIp acetylated at Lysine 676 is a diagnostic marker of severity in SARS-CoV-2 pneumonia
Sacchi et al. (83)	Polymorphonuclear myeloid-derived suppressor cells inhibit T-cell IFN-λ production during SARS-CoV-2 peptide stimulation through TGFβ mechanisms
Ghazavi et al. (82)	Serum TGFβ levels increase with increasing disease severity in patients with COVID-19
Kvedaraite et al. (99)	Serum TGFβ levels correlate with circulating monocyte phenotype in severe COVID-19
Ferreira-Gomes et al. (80)	SARS-CoV-2 triggers an immune response instructed by TGFβ, including IgA class switching
D’Agnillo et al. (95)	TGFβ1 staining co-localizes with PAI-1 in pulmonary blood vessels and developing fibrotic lesions in COVID-19 lung tissue
Witkowski et al. (78)	Serum levels of TGFβ peak during the first 2 weeks of severe COVID-19 infection and inhibit NK cell function
Colarusso et al. (97)	Higher TGFβ levels predict an increased relative risk of lung fibrosis-like changes in post-COVID patients
Vaz de Paula et al. (96)	Immuno-expression of TGFβ1 is increased in lung tissue of patients who died of COVID-19
Dinnon et al. (109)	Transcriptomic analysis of diseased alveolar regions shows up-regulated signaling by the TGFβ receptor complex in mouse-adapted SARS-CoV-2
Colarusso et al. (98)	TGFβ has higher stimulated release in post-COVID patients with signs of lung fibrosis on chest computed tomography scans

All listed studies are cited in the references section. TGF-β, transforming growth factor beta; LLC, large latent complex; NK, natural killer; PAI-I, plasminogen activator inhibitor; TGFβIp, TGFβ induced protein.

### TGF-β pathway inhibitors may ameliorate TGF-β driven thrombosis and immune dysregulation in COVID-19

Given the substantial evidence that SARS-CoV-2 stimulates host TGF-β pathways, TGF-β antagonism arises as a potential therapeutic target for improving outcomes during COVID-19 illness. Such an approach was suggested by multiple investigators early in the COVID-19 pandemic (80, 108, 116, 117). Over a dozen TGF-β inhibitors (TGF-βi) have been evaluated in human clinical trials of cancer and inflammatory diseases. Thus given the evidence presented above, the TGF-βi space is ripe for immediate repurposing for animal “proof of principle” studies and subsequent clinical trials of COVID-19 (Table 3; 118). A number of robust animal models exist for COVID-19, allowing rapid assessment of candidate TGF-βi for efficacy and safety to identify agents worth promoting to human clinical trials (119–122).

**TABLE 3:** Transforming growth factor-beta (TGF-β) pathway inhibitors (approved, currently in clinical trials, or preclinical) potentially relevant to COVID-19 prevention or treatment.

Drug	Manufacturer	Route	Mechanism	Status diseases	Clinical trials	Clinical trial stage/Findings (as of 8/2022)
GSK3008348	Glaxo	Inhaled	ανβ6 integrin inhibitor	Investigational lung fibrosis	NCT02612051 NCT03069989 NCT02612051	Phase I Ongoing or completed/Unpublished
PLN-74809	Pliant Therapeutics	Oral	Small molecule ανβ6/ανβ1 integrin inhibitor	Investigational lung fibrosis cholangitis	NCT04072315 NCT04396756 NCT04480840	Phase IIa Ongoing or completed/Unpublished
MORF-720	Morphic (AbbVie)	Oral	Small molecule ανβ6, 4β7 integrin inhibitors	Pre-clinical fibrosis cancer	NA	NA
LY2157299 (Galunisertib)	Eli Lilly	Oral	Small molecule, TGFβRIi	Investigational cancer	Of 25 trials: NCT02304419 NCT02008318	Phase 1–3 Well-tolerated, benefit seen in phase 2 studies
LY573636 (Tasisulam)	Eli Lilly	IV	Small molecule, TGFβRIi; long half-life	Investigational cancer	Of 13 studies: NCT00992225	Phase 2 Hepatic and marrow toxicity
LY3200882	Eli Lilly	Oral	Small molecule, TGFβRIi	Investigational cancer	Of 4 trials: NCT04031872 NCT02937272	Phase 1–2 Well-tolerated
Vactosertib (TEW7197)	Bristol Myers Squibb (Forbius)	Oral	Small molecule, TGFβRIi	Investigational cancer	Of 17 trials: NCT03074006 NCT04515979	Phase 2 Ongoing or completed/Unpublished
SRI-35241	Southern Research	IV	Small molecule Inhibits activation of latent TGFβ	Preclinical fibrosis	Preclinical	Preclinical
Epigallocatechin Gallate (ECGC)	Multiple producers	Oral	Green tree extract Catechin Kinase inhibitor	Investigational lung fibrosis many diseases	Of 104 trials: NCT03928847 NCT03928847	Marketed as over-the-counter supplement (FDA approval not required)
AVID200	Bristol Myers Squibb (Forbius)	IV	TGFβ 1/3 trap	Investigational cancer skin fibrosis myelofibrosis	NCT03831438 NCT03834662 NCT03895112	Phase 2 Well-tolerated, biologic effects demonstrated *in vivo*
Luspatercept	Acceleron (Merck)	SQ	TGFβ trap (ACtRIIb)	FDA-approved myelofibrosis thalassemia	Of 25 trials: NCT02604433 NCT04717414	Marketed after phase 3 trials Improves hemoglobin levels Other indications being tested in clinical trials
Sotatercept (ACE-001)	Acceleron (Merck)	SQ	TGFβ trap (ACtRIIa)	Investigational pulmonary hypertension	Of 21 trials: NCT03496207 NCT04896008	Phase 2
Bintrafusp-α (M7824)	EMD Serrano (Merck-GSK)	IV	Bifunctional Ab: TGFβ-trap, aPDL1	Investigational cancer	Of 46 trials: NCT02517398 NCT04727541	Phase 1–2; ongoing or completed Tolerable (rash, pruritis)
Dalutrafusp-α (GS-1423)	Gilead	IV	Bifunctional Ab: aCD73, TGFβ-Trap	Investigational cancer	NCT03954704	Phase 1, terminated
Trabedersen AP12009 OT-101	Autotelic (Isarna)	IV topical	Antisense RNA to TGFβ2	Investigational cancer COVID-19 myopia	NCT00844064 NCT00431561 NCT00761280 NCT04801017	Phase 2–3 Ongoing or completed/Unpublished
ISTH0036	Isarna Therapeutics	Eye	Antisense RNA to TGFβ1	Investigational glaucoma AMD	NCT02406833	Phase 2, Ongoing
TRK-250	Toray Industries	Inhaled	Antisense RNA to TGFβ1	Investigational Lung fibrosis	NCT03727802	Phase 1, Ongoing
XOMA089	Novartis	IV/SQ	TGFβ 1-3 Ab (all isoforms)	Preclinical	NA	NA
LY2382770	Eli Lilly	SQ	TGFβ1 Ab	Investigational kidney disease	NCT01113801	Phase 2, completed No clinical benefit to date Well-tolerated
Fresolimumab (GC1008)	Sanofi	IV	TGFβ 1-3 Ab (to all isoforms)	Investigational cancer fibrosis	Of 12 trials: NCT00125385 NCT01665391	Phase 2; completed No clinical benefit to date Skin toxicity
SRK-181	Gilead (Scholar-Rock)	IV	Latent-TGFβ1 Ab	Investigational cancer	NCT04291079	Phase 1, Ongoing
NA	Merck (Tilos)	IV	Anti-LAP integrin Ab	Pre-clinical cancer	NA	NA
BG00011 (STX100)	Biogen	SQ	anti-αvβ6 integrin Ab	Investigational Lung fibrosis	NCT01371305	Phase 2, unpublished Trials stopped early 2017
VTX-001	Venn Therapeutics	IV/SQ	anti-αvβ6 integrin Ab	Pre-clinical fibrosis	None to date	NA

Some pre-clinical agents and other agents with halted development are not shown. Only one drug is FDA-approved (luspatercept), and only one drug is currently in COVID-19 clinical trials (trabedersen). Ab, antibody; ACtRIIa, activin receptor IIa; ACtRIIb, activin receptor IIb; AMD, adult macular degeneration; aPDL1, anti-programmed death ligand 1; IV, intravenous; LAP, latency activating peptide; NA, not applicable; SQ, subcutaneous; TGF-β, transforming growth factor beta; TGFβIR, TGF-β receptor I, also known as ALK5; TGFβR2 trap, Fc-based compound containing copies of TGF-β receptor II; TGFβRIi, TGF-β receptor I inhibitor.

To date, the majority of TGF-βi have failed to benefit primary outcomes in phase I-II clinical trials of various cancers and inflammatory diseases (123–127). For some agents, recognition of concerning adverse events limited further development (127, 128). None of these studies evaluated thrombosis as an outcome. These existing TGF-βi target multiple points in the TGF-β pathway (Table 3). TGF-β receptor inhibitors are typically small molecule oral drugs that inhibit intracellular TGF-β receptor I (TGFBRI, also known as ALK5) or TGF-β receptor II (TGFBRII) kinase activity, including catechin polyphenol derivatives of green tea extracts (52, 126). Antibodies to TGF-β ligand (both to individual and to all TGF-β isoforms) also exist, as do ligand traps (124, 129–131). Agents targeted at preventing L-TGF-β activation such as integrin inhibitors have already entered clinical trials of inflammatory diseases (125), and antibodies and small molecules blocking L-TGF-β are in preclinical development (132, 133). Angiotensin converting enzyme inhibitors and angiotensin-II receptor blockers such as losartan has been evaluated in clinical trials of COVID-19 (NCT04312009 and NCT04312009) given their low cost, worldwide availability as approved drugs for hypertension and heart failure, and their potential modulation of surface expression of the ACE2 receptor where the SARS-CoV-2 spike protein binds for its cellular uptake route. These agents are also known to have off-target effects *via* decreased TGF-β signaling through inhibiting synthesis of TSP1 and other mediators that increase active TGF-β (134). They have been used to slow TGF-β mediated thoracic aortic aneurysm progression in Marfan’s disease with evidence for improved outcomes (135). To date, losartan has not been shown to improve outcomes in COVID-19 (136). As these agents are not specifically directed at TGF-β, they are not a subject of this review.

Antisense RNA agents targeting TGF-β1 or TGF-β2 are also in development or in clinical trials (137). Notably, while only one of these agents is FDA-approved, in most cases these agents could undergo a rapid production phase as most have already completed phase I trials. These would be reasonable agents to test in animal models of COVID-19, selecting promising agents for further evaluation in human trials. One TGF-βi, luspatercept, is an FDA-approved TGF-β “trap” that is already marketed worldwide in a subcutaneous formulation to decrease red cell transfusion needs in thalassemia. In thalassemia, marrow production of red blood cells is inhibited by TGF-β (129). Pirfenidone and nintedanib are an antifibrotic agents that have anti-TGF-β properties and are approved in many countries for the treatment of idiopathic pulmonary fibrosis. Pirfenidone works in part by inhibiting furin, an enzyme that activates TGF-β from its precursor form. Furin is also necessary for SARS-CoV-2 internalization (138). A phase 2 trial of pirfenidone in 146 inpatients with acute COVID-19 (NCT04282902) did not meet its primary endpoint for change in pulmonary-related quality of life scoring at week 4, but improvements in secondary outcomes such as decreased proinflammatory cytokines, decreased coagulation biomarkers, and decreased hospital days were reported (139). Pirfenidone is currently in a phase II clinical trial for post-COVID-19 pulmonary fibrosis (NCT04607928). Nintedanib is also being evaluated in post-COVID fibrosis in several phase III-IV trials (NCT04541680 and NCT04619680). To date, only one clinical trial of a specific TGF-βi for use during COVID-19 illness has begun and is ongoing. This trial tests the effect of trabedersen, an antisense RNA for TGF-β2 (137). Trabedersen has been repurposed for evaluation in COVID-19, a decade after an initial clinical trial in brain tumors did not show improved outcomes (140). The ongoing phase 2 double blind placebo-controlled trial in South America of hospitalized severe COVID-19 patients involves a 7-day intravenous infusion of trabedersen or placebo in combination with the oral anti-malarial drug artemisinin (which binds to TGF-βR2) (141); results have not been published (NCT04801017).

Transforming growth factor-beta (TGF-β) inhibition will also have direct or indirect effects on other cytokines that should on balance be beneficial during COVID-19 illness. TGF-β1 drives proinflammatory Th17 cell production (38), and Th17 cell number and activity (including IL-17 and IL-23 secretion) are increased during COVID-19 illness (86, 142). TGF-β1 also indirectly upregulates HIF-1α induced genes *via* decreased expression of PHD2, which leads to an increase in intracellular HIF-1α levels (since PHD2 normally leads to hydroxylation and ubiquitination/destruction of HIF-1α) (45). Thus, HIF-1α driven genes such as vascular endothelial growth factor, erythropoietin, heme oxygenase-1, and glucose transporter 1 would be expected to decrease with TGF-βi.

### Scope and limitations of using TGF-β inhibitors and antisense RNA agents

The best timing of administration of TGF-βi in this paradigm remains unclear. Existing data suggests that SARS-CoV-2 exploitation of TGF-β and related immune evasion most likely occurs during early infection (week 1) when a milder illness is present. During this early stage, an immune reconstitution driven by TGF-βi administration would strengthen anti-viral immunity and be less likely to have adverse effects. However, even after the onset of fever and a proinflammatory state or cytokine storm in more severe COVID-19 cases (correlating with immune recognition of infection, typically week 2 post-infection), evidence of ongoing TGF-β activity persists–such as IgA antibody class switching, high TGF-β blood levels, Th17 pathway activation, and the onset of lung fibrosis (54, 80, 86, 97, 105, 143). Thus, TGF-β pathway activity may remain high over most of the illness course, particularly in severe cases and during lung fibrosis. During these later inflammatory stages, an immune reconstitution driven by TGF-βi administration could trigger adverse inflammatory effects. For example, in one study of cancer patients given an antibody to TGF-βRII, a cytokine release syndrome developed that limited further drug development and suggested that immune stimulation occurred with this agent (144). However, such adverse events have been rare with TGF-βi and may be ameliorated in COVID-19 by concomitant therapies such as steroids and anti-inflammatory agents that are already given in hospitalized patients with severe illness requiring oxygen therapy (84, 103). Current studies are insufficient to implicate TGF-β in the “long covid” syndrome, also known as the post-acute syndrome of COVID (PASC). However, Colarusso et al. have demonstrated elevated blood levels of TGF-β1 and stimulated blood monocyte release of excessive TGF-β1 in patients with persistent lung abnormalities on computed tomography scans months after clearance of SARS-CoV-2 (97, 98, 145). If clinical trials of TGF-βi are shown to be effective in acute COVID-19, it will be important to assess their effect on PASC.

Any salutary effects of TGF-βi on COVID-19 induced thrombosis would have to be additional to those already proven for the standard therapeutic anticoagulant heparin (92). Moreover, due to TGF-β’s pluripotent effects on inflammatory and immune mediators, separating out specific TGF-βi effects on thrombosis will be difficult in the greater context of its more direct effects on immune function and inflammation. Moreover, unexpected side effects could occur with TGF-βi given the panoply of genes and processes that TGF-β regulates.

## Summary

Thrombosis, inflammation, and immune dysregulation are all key events that contribute to microvascular injury and adverse outcomes during SARS-CoV-2 infection. Increasing evidence suggests that activation of the TGF-β pathway is a prominent feature of the microvascular clotting and inflammatory injury characteristic of SARS-CoV-2 infection. Given the substantial evolution seen in other viruses and pathogens where enhancement of their fitness is achieved by TGF-β hijacking, a paradigm exists where SARS-CoV-2 elicits initial immune evasion by increasing activation of host TGF-β. Ongoing TGF-β activation then promotes collateral downstream adverse effects during the inflammatory phase of COVID-19 such as continued immune dysregulation, inflammatory injury including Th17 pathway activation, and fibrosis. These reports suggest that TGF-β pathway inhibitors may be a useful approach to improve outcomes in SARS-CoV-2 infection. One such inhibitor is already being tested in a clinical trial of COVID-19, one inhibitor is FDA-approved for another illness, and over a dozen inhibitors are available for repurposing after unsuccessful or ongoing clinical trials in cancer and inflammatory diseases. Evaluation of these inhibitors in animal models of COVID-19 is highly feasible and will be an important first step for “proof of concept” before clinical trials in humans with SARS-CoV-2 infection.

## Author contributions

EC and JM created the original figures. All authors wrote the manuscript, edited and contributed to tables and figures, and approved the final version of the manuscript.

## References

[1] Morikawa M Derynck R Miyazono K. TGF-beta and the TGF-beta family: context-dependent roles in cell and tissue physiology. *Cold Spring Harb Perspect Biol.* (2016) 8:a021873. 10.1101/cshperspect.a021873 PMC4852809 27141051

[2] Kulkarni A Huh C Becker D Geiser A Lyght M Flanders K Transforming growth factor beta 1 null mutation in mice causes excessive inflammatory response and early death. *Proc Natl Acad Sci U.S.A.* (1993) 90:770–4. 8421714 10.1073/pnas.90.2.770 PMC45747

[3] Sanford L Ormsby I Gittenberger-de Groot A Sariola H Friedman R Boivin G TGFbeta2 knockout mice have multiple developmental defects that are non-overlapping with other TGFbeta knockout phenotypes. *Development.* (1997) 124:2659–70. 10.1242/dev.124.13.2659 9217007 PMC3850286

[4] Kaartinen V Voncken J Shuler C Warburton D Bu D Heisterkamp N Abnormal lung development and cleft palate in mice lacking TGF-beta 3 indicates defects of epithelial-mesenchymal interaction. *Nat Genet.* (1995) 11:415–21. 10.1038/ng1295-415 7493022

[5] Yoshinaga K Obata H Jurukovski V Mazzieri R Chen Y Zilberberg L Perturbation of transforming growth factor (TGF)-beta1 association with latent TGF-beta binding protein yields inflammation and tumors. *Proc Natl Acad Sci U.S.A.* (2008) 105:18758–63. 10.1073/pnas.0805411105 19022904 PMC2596235

[6] Schultz-Cherry S Ribeiro S Gentry L Murphy-Ullrich J. Thrombospondin binds and activates the small and large forms of latent transforming growth factor-beta in a chemically defined system. *J Biol Chem.* (1994) 269:26775–82. 7929413

[7] Young G Murphy-Ullrich J. Molecular interactions that confer latency to transforming growth factor-beta. *J Biol Chem.* (2004) 279:38032–9. 15208302 10.1074/jbc.M405658200

[8] Mu D Cambier S Fjellbirkeland L Baron J Munger J Kawakatsu H The integrin alpha(v)beta8 mediates epithelial homeostasis through MT1-MMP-dependent activation of TGF-beta1. *J Cell Biol.* (2002) 157:493–507. 10.1083/jcb.200109100 11970960 PMC2173277

[9] Lu M Munger J Steadele M Busald C Tellier M Schnapp L. Integrin alpha8beta1 mediates adhesion to LAP-TGFbeta1. *J Cell Sci.* (2002) 115:4641–8. 10.1242/jcs.00145 12415008

[10] Schultz-Cherry S Chen H Mosher D Misenheimer T Krutzsch H Roberts D Regulation of transforming growth factor-beta activation by discrete sequences of thrombospondin 1. *J Biol Chem.* (1995) 270:7304–10. 10.1074/jbc.270.13.7304 7706271

[11] Munger J Huang X Kawakatsu H Griffiths M Dalton S Wu J The integrin alpha v beta 6 binds and activates latent TGF beta 1: a mechanism for regulating pulmonary inflammation and fibrosis. *Cell.* (1999) 96:319–28. 10025398 10.1016/s0092-8674(00)80545-0

[12] Coker R Laurent G Shahzeidi S Hernandez-Rodriguez N Pantelidis P du Bois R Diverse cellular TGF-beta 1 and TGF-beta 3 gene expression in normal human and murine lung. *Eur Respir J.* (1996) 9:2501–7. 10.1183/09031936.96.09122501 8980960

[13] Brown P Wakefield L Levinson A Sporn M. Physicochemical activation of recombinant latent transforming growth factor-beta’s 1, 2, and 3. *Growth Factors.* (1990) 3:35–43. 10.3109/08977199009037500 2200452

[14] Travis M Sheppard D. TGF-beta activation and function in immunity. *Annu Rev Immunol.* (2014) 32:51–82. 24313777 10.1146/annurev-immunol-032713-120257 PMC4010192

[15] Assoian R Komoriya A Meyers C Miller D Sporn M. Transforming growth factor-beta in human platelets. Identification of a major storage site, purification, and characterization. *J Biol Chem.* (1983) 258:7155–60. 6602130

[16] Crawford S Stellmach V Murphy-Ullrich J Ribeiro S Lawler J Hynes R Thrombospondin-1 is a major activator of TGF-beta1 in vivo. *Cell.* (1998) 93:1159–70. 10.1016/s0092-8674(00)81460-9 9657149

[17] Maloney J Narasimhan J Biller J. Decreased TGF-beta1 and VEGF release in cystic fibrosis platelets: further evidence for platelet defects in cystic fibrosis. *Lung.* (2016) 194:791–8. 10.1007/s00408-016-9925-9 27423781 PMC5033711

[18] Meyer A Wang W Qu J Croft L Degen J Coller B Platelet TGF-beta1 contributions to plasma TGF-beta1, cardiac fibrosis, and systolic dysfunction in a mouse model of pressure overload. *Blood.* (2012) 119:1064–74. 10.1182/blood-2011-09-377648 22134166 PMC3271718

[19] Grainger D Wakefield L Bethell H Farndale R Metcalfe J. Release and activation of platelet latent TGF-beta in blood clots during dissolution with plasmin. *Nat Med.* (1995) 1:932–7. 10.1038/nm0995-932 7585220

[20] Blakytny R Ludlow A Martin G Ireland G Lund L Ferguson M Latent TGF-beta1 activation by platelets. *J Cell Physiol.* (2004) 199:67–76. 14978736 10.1002/jcp.10454

[21] Ahamed J Burg N Yoshinaga K Janczak C Rifkin D Coller B. In vitro and in vivo evidence for shear-induced activation of latent transforming growth factor-beta1. *Blood.* (2008) 112:3650–60. 10.1182/blood-2008-04-151753 18544680 PMC2572792

[22] Karolczak K Watala C. Blood platelets as an important but underrated circulating source of TGFbeta. *Int J Mol Sci.* (2021) 22:4492. 10.3390/ijms22094492 33925804 PMC8123509

[23] Huber D Fontana A Bodmer S. Activation of human platelet-derived latent transforming growth factor-beta 1 by human glioblastoma cells. Comparison with proteolytic and glycosidic enzymes. *Biochem J.* (1991) 277(Pt 1):165–73. 10.1042/bj2770165 1830205 PMC1151206

[24] Lev P Salim J Marta R Osorio M Goette N Molinas F. Platelets possess functional TGF-beta receptors and Smad2 protein. *Platelets.* (2007) 18:35–42. 10.1080/09537100600800743 17365852

[25] Hoying J Yin M Diebold R Ormsby I Becker A Doetschman T. Transforming growth factor beta1 enhances platelet aggregation through a non-transcriptional effect on the fibrinogen receptor. *J Biol Chem.* (1999) 274:31008–13. 10.1074/jbc.274.43.31008 10521498

[26] Jablonska E Markart P Zakrzewicz D Preissner K Wygrecka M. Transforming growth factor-beta1 induces expression of human coagulation factor XII via Smad3 and JNK signaling pathways in human lung fibroblasts. *J Biol Chem.* (2010) 285:11638–51. 10.1074/jbc.M109.045963 20142324 PMC2857041

[27] Saito M Ichikawa J Ando T Schoenecker J Ohba T Koyama K Platelet-derived TGF-beta induces tissue factor expression via the Smad3 pathway in osteosarcoma cells. *J Bone Miner Res.* (2018) 33:2048–58. 10.1002/jbmr.3537 29949655

[28] Dong C Zhu S Wang T Yoon W Goldschmidt-Clermont P. Upregulation of PAI-1 is mediated through TGF-beta/Smad pathway in transplant arteriopathy. *J Heart Lung Transplant.* (2002) 21:999–1008. 12231371 10.1016/s1053-2498(02)00403-5

[29] Stukalov A Girault V Grass V Karayel O Bergant V Urban C Multilevel proteomics reveals host perturbations by SARS-CoV-2 and SARS-CoV. *Nature.* (2021) 594:246–52. 10.1038/s41586-021-03493-4 33845483

[30] Kim H Kim P Bae S Son H Thoudam D Kim J Transforming growth factor-beta-induced protein (TGFBIp/beta ig-h3) activates platelets and promotes thrombogenesis. *Blood.* (2009) 114:5206–15. 10.1182/blood-2009-03-212415 19738031

[31] Tran D Andersson J Wang R Ramsey H Unutmaz D Shevach E. GARP (LRRC32) is essential for the surface expression of latent TGF-beta on platelets and activated FOXP3+ regulatory T cells. *Proc Natl Acad Sci U.S.A.* (2009) 106:13445–50. 10.1073/pnas.0901944106 19651619 PMC2726354

[32] Lawler J Sunday M Thibert V Duquette M George E Rayburn H Thrombospondin-1 is required for normal murine pulmonary homeostasis and its absence causes pneumonia. *J Clin Invest.* (1998) 101:982–92. 10.1172/JCI1684 9486968 PMC508649

[33] Li M Sanjabi S Flavell R. Transforming growth factor-beta controls development, homeostasis, and tolerance of T cells by regulatory T cell-dependent and -independent mechanisms. *Immunity.* (2006) 25:455–71. 10.1016/j.immuni.2006.07.011 16973386

[34] Barral A Barral-Netto M Yong E Brownell C Twardzik D Reed S. Transforming growth factor beta as a virulence mechanism for Leishmania braziliensis. *Proc Natl Acad Sci U.S.A.* (1993) 90:3442–6. 10.1073/pnas.90.8.3442 7682701 PMC46316

[35] Sanjabi S Oh S Li M. Regulation of the immune response by TGF-beta: from conception to autoimmunity and infection. *Cold Spring Harb Perspect Biol.* (2017) 9:a022236. 10.1101/cshperspect.a022236 PMC5453394 28108486

[36] Batlle E Massague J. Transforming growth factor-beta signaling in immunity and cancer. *Immunity.* (2019) 50:924–40. 30995507 10.1016/j.immuni.2019.03.024 PMC7507121

[37] Wan Y Flavell R. ‘Yin-Yang’ functions of transforming growth factor-beta and T regulatory cells in immune regulation. *Immunol Rev.* (2007) 220:199–213. 10.1111/j.1600-065X.2007.00565.x 17979848 PMC2614905

[38] Gutcher I Donkor M Ma Q Rudensky A Flavell R Li M. Autocrine transforming growth factor-beta1 promotes in vivo Th17 cell differentiation. *Immunity.* (2011) 34:396–408. 10.1016/j.immuni.2011.03.005 21435587 PMC3690311

[39] Voo K Wang Y Santori F Boggiano C Wang Y Arima K Identification of IL-17-producing FOXP3+ regulatory T cells in humans. *Proc Natl Acad Sci U.S.A.* (2009) 106:4793–8. 19273860 10.1073/pnas.0900408106 PMC2653560

[40] Tsai H Velichko S Hung L Wu R. IL-17A and Th17 cells in lung inflammation: an update on the role of Th17 cell differentiation and IL-17R signaling in host defense against infection. *Clin Dev Immunol.* (2013) 2013:267971. 10.1155/2013/267971 23956759 PMC3730142

[41] Lu Y Sun K Guo S Wang J Li A Rong X Early warning indicators of severe COVID-19: a single-center study of cases from Shanghai, China. *Front Med (Lausanne).* (2020) 7:432. 10.3389/fmed.2020.00432 32766268 PMC7379420

[42] Peters D Vadasz I Wujak L Wygrecka M Olschewski A Becker C TGF-beta directs trafficking of the epithelial sodium channel ENaC which has implications for ion and fluid transport in acute lung injury. *Proc Natl Acad Sci U.S.A.* (2014) 111:E374–83. 10.1073/pnas.1306798111 24324142 PMC3903252

[43] Pittet J Griffiths M Geiser T Kaminski N Dalton S Huang X TGF-beta is a critical mediator of acute lung injury. *J Clin Invest.* (2001) 107:1537–44. 11413161 10.1172/JCI11963 PMC200192

[44] Yan F Wang Y Wu X Peshavariya H Dusting G Zhang M Nox4 and redox signaling mediate TGF-beta-induced endothelial cell apoptosis and phenotypic switch. *Cell Death Dis.* (2014) 5:e1010. 10.1038/cddis.2013.551 24457954 PMC4040700

[45] McMahon S Charbonneau M Grandmont S Richard D Dubois C. Transforming growth factor beta1 induces hypoxia-inducible factor-1 stabilization through selective inhibition of PHD2 expression. *J Biol Chem.* (2006) 281:24171–81. 10.1074/jbc.M604507200 16815840

[46] Budinger G Chandel N Donnelly H Eisenbart J Oberoi M Jain M. Active transforming growth factor-beta1 activates the procollagen I promoter in patients with acute lung injury. *Intensive Care Med.* (2005) 31:121–8. 10.1007/s00134-004-2503-2 15565360 PMC7095267

[47] Fahy R Lichtenberger F McKeegan C Nuovo G Marsh C Wewers M. The acute respiratory distress syndrome: a role for transforming growth factor-beta 1. *Am J Respir Cell Mol Biol.* (2003) 28:499–503. 12654639 10.1165/rcmb.2002-0092OC

[48] Jenkins R Su X Su G Scotton C Camerer E Laurent G Ligation of protease-activated receptor 1 enhances alpha(v)beta6 integrin-dependent TGF-beta activation and promotes acute lung injury. *J Clin Invest.* (2006) 116:1606–14. 10.1172/JCI27183 16710477 PMC1462943

[49] Forel J Guervilly C Farnarier C Donati S Hraiech S Persico N Transforming growth factor-beta1 in predicting early lung fibroproliferation in patients with acute respiratory distress syndrome. *PLoS One.* (2018) 13:e0206105. 10.1371/journal.pone.0206105 30395619 PMC6218031

[50] Khalil N Parekh T O’Connor R Antman N Kepron W Yehaulaeshet T Regulation of the effects of TGF-beta 1 by activation of latent TGF-beta 1 and differential expression of TGF-beta receptors (T beta R-I and T beta R-II) in idiopathic pulmonary fibrosis. *Thorax.* (2001) 56:907–15. 10.1136/thorax.56.12.907 11713352 PMC1745982

[51] Ask K Bonniaud P Maass K Eickelberg O Margetts P Warburton D Progressive pulmonary fibrosis is mediated by TGF-beta isoform 1 but not TGF-beta3. *Int J Biochem Cell Biol.* (2008) 40:484–95. 17931953 10.1016/j.biocel.2007.08.016 PMC2350199

[52] Chapman H Wei Y Montas G Leong D Golden J Trinh B Reversal of TGFbeta1-driven profibrotic state in patients with pulmonary fibrosis. *N Engl J Med.* (2020) 382:1068–70. 10.1056/NEJMc1915189 32160670 PMC7297220

[53] Neubauer E Poole A Neubauer P Detournay O Tan K Davy S A diverse host thrombospondin-type-1 repeat protein repertoire promotes symbiont colonization during establishment of cnidarian-dinoflagellate symbiosis. *Elife.* (2017) 6:e24494. 10.7554/eLife.24494 28481198 PMC5446238

[54] Sterlin D Mathian A Miyara M Mohr A Anna F Claer L IgA dominates the early neutralizing antibody response to SARS-CoV-2. *Sci Transl Med.* (2021) 13:eabd2223. 10.1126/scitranslmed.abd2223 PMC7857408 33288662

[55] Presser L Haskett A Waris G. Hepatitis C virus-induced furin and thrombospondin-1 activate TGF-beta1: role of TGF-beta1 in HCV replication. *Virology.* (2011) 412:284–96. 10.1016/j.virol.2010.12.051 21296375 PMC3073624

[56] Simmons K Nde P Kleshchenko Y Lima M Villalta F. Stable RNA interference of host thrombospondin-1 blocks Trypanosoma cruzi infection. *FEBS Lett.* (2006) 580:2365–70. 10.1016/j.febslet.2006.03.054 16616140

[57] Schultz-Cherry S Hinshaw V. Influenza virus neuraminidase activates latent transforming growth factor beta. *J Virol.* (1996) 70:8624–9. 8970987 10.1128/jvi.70.12.8624-8629.1996 PMC190955

[58] Zhao X Nicholls J Chen Y. Severe acute respiratory syndrome-associated coronavirus nucleocapsid protein interacts with Smad3 and modulates transforming growth factor-beta signaling. *J Biol Chem.* (2008) 283:3272–80. 10.1074/jbc.M708033200 18055455 PMC8740907

[59] Omer F de Souza J Corran P Sultan A Riley E. Activation of transforming growth factor beta by malaria parasite-derived metalloproteinases and a thrombospondin-like molecule. *J Exp Med.* (2003) 198:1817–27. 10.1084/jem.20030713 14676296 PMC2194152

[60] Putignani L Possenti A Cherchi S Pozio E Crisanti A Spano F. The thrombospondin-related protein CpMIC1 (CpTSP8) belongs to the repertoire of micronemal proteins of Cryptosporidium parvum. *Mol Biochem Parasitol.* (2008) 157:98–101. 10.1016/j.molbiopara.2007.09.004 17981348

[61] Kumar R Mickael C Kassa B Gebreab L Robinson J Koyanagi D TGF-beta activation by bone marrow-derived thrombospondin-1 causes Schistosoma- and hypoxia-induced pulmonary hypertension. *Nat Commun.* (2017) 8:15494. 10.1038/ncomms15494 28555642 PMC5459967

[62] Kumar R Mickael C Kassa B Sanders L Hernandez-Saavedra D Koyanagi D Interstitial macrophage-derived thrombospondin-1 contributes to hypoxia-induced pulmonary hypertension. *Cardiovasc Res.* (2019) 116:2021–30. 10.1093/cvr/cvz304 31710666 PMC7519884

[63] Denney L Branchett W Gregory L Oliver R Lloyd C. Epithelial-derived TGF-beta1 acts as a pro-viral factor in the lung during influenza A infection. *Mucosal Immunol.* (2018) 11:523–35. 10.1038/mi.2017.77 29067998 PMC5797694

[64] Bouhaddou M Memon D Meyer B White K Rezelj V Correa Marrero M The global phosphorylation landscape of SARS-CoV-2 infection. *Cell.* (2020) 182:685–712.e19. 32645325 10.1016/j.cell.2020.06.034 PMC7321036

[65] Li Q Wang Y Sun Q Knopf J Herrmann M Lin L Immune response in COVID-19: what is next? *Cell Death Differ.* (2022) 29:1107–22. 35581387 10.1038/s41418-022-01015-x PMC9110941

[66] Wilk A Rustagi A Zhao N Roque J Martinez-Colon G McKechnie J A single-cell atlas of the peripheral immune response in patients with severe COVID-19. *Nat Med.* (2020) 26:1070–6. 32514174 10.1038/s41591-020-0944-y PMC7382903

[67] Han H Ma Q Li C Liu R Zhao L Wang W Profiling serum cytokines in COVID-19 patients reveals IL-6 and IL-10 are disease severity predictors. *Emerg Microbes Infect.* (2020) 9:1123–30. 32475230 10.1080/22221751.2020.1770129 PMC7473317

[68] Grant R Morales-Nebreda L Markov N Swaminathan S Querrey M Guzman E Circuits between infected macrophages and T cells in SARS-CoV-2 pneumonia. *Nature.* (2021) 590:635–41. 33429418 10.1038/s41586-020-03148-w PMC7987233

[69] Magro C Mulvey J Berlin D Nuovo G Salvatore S Harp J Complement associated microvascular injury and thrombosis in the pathogenesis of severe COVID-19 infection: a report of five cases. *Transl Res.* (2020) 220:1–13. 10.1016/j.trsl.2020.04.007 32299776 PMC7158248

[70] Middleton E He X Denorme F Campbell R Ng D Salvatore S Neutrophil extracellular traps contribute to immunothrombosis in COVID-19 acute respiratory distress syndrome. *Blood.* (2020) 136:1169–79. 32597954 10.1182/blood.2020007008 PMC7472714

[71] Peiris J Yuen K Osterhaus A Stohr K. The severe acute respiratory syndrome. *N Engl J Med.* (2003) 349:2431–41. 14681510 10.1056/NEJMra032498

[72] Kaminski N Allard J Pittet J Zuo F Griffiths M Morris D Global analysis of gene expression in pulmonary fibrosis reveals distinct programs regulating lung inflammation and fibrosis. *Proc Natl Acad Sci U.S.A.* (2000) 97:1778–83. 10677534 10.1073/pnas.97.4.1778 PMC26512

[73] Li S Wang C Jou Y Yang T Huang S Wan L SARS coronavirus papain-like protease induces Egr-1-dependent up-regulation of TGF-beta1 via ROS/p38 MAPK/STAT3 pathway. *Sci Rep.* (2016) 6:25754. 10.1038/srep25754 27173006 PMC4865725

[74] Li S Yang T Wan L Lin Y Tsai F Lai C Correlation between TGF-beta1 expression and proteomic profiling induced by severe acute respiratory syndrome coronavirus papain-like protease. *Proteomics.* (2012) 12:3193–205. 10.1002/pmic.201200225 22936401 PMC7168038

[75] He L Ding Y Zhang Q Che X He Y Shen H Expression of elevated levels of pro-inflammatory cytokines in SARS-CoV-infected ACE2+ cells in SARS patients: relation to the acute lung injury and pathogenesis of SARS. *J Pathol.* (2006) 210:288–97. 10.1002/path.2067 17031779 PMC7167655

[76] Zhang Y Li J Zhan Y Wu L Yu X Zhang W Analysis of serum cytokines in patients with severe acute respiratory syndrome. *Infect Immun.* (2004) 72:4410–5. 15271897 10.1128/IAI.72.8.4410-4415.2004 PMC470699

[77] Mann E Menon M Knight S Konkel J Jagger C Shaw T Longitudinal immune profiling reveals key myeloid signatures associated with COVID-19. *Sci Immunol.* (2020) 5:eabd6197. 10.1126/sciimmunol.abd6197 PMC7857390 32943497

[78] Witkowski M Tizian C Ferreira-Gomes M Niemeyer D Jones T Heinrich F Untimely TGFbeta responses in COVID-19 limit antiviral functions of NK cells. *Nature.* (2021) 600:295–301. 10.1038/s41586-021-04142-6 34695836

[79] Park H Kim H Kim H Yoo Y Shin H Choi E Acetylated K676 TGFBIp as a severity diagnostic blood biomarker for SARS-CoV-2 pneumonia. *Sci Adv.* (2020) 6:eabc1564. 10.1126/sciadv.abc1564 32937590 PMC10715714

[80] Ferreira-Gomes M Kruglov A Durek P Heinrich F Tizian C Heinz G SARS-CoV-2 in severe COVID-19 induces a TGF-beta-dominated chronic immune response that does not target itself. *Nat Commun.* (2021) 12:1961. 10.1038/s41467-021-22210-3 33785765 PMC8010106

[81] Yu H Sun B Fang Z Zhao J Liu X Li Y Distinct features of SARS-CoV-2-specific IgA response in COVID-19 patients. *Eur Respir J.* (2020) 56:2001526. 10.1183/13993003.01526-2020 32398307 PMC7236821

[82] Ghazavi A Ganji A Keshavarzian N Rabiemajd S Mosayebi G. Cytokine profile and disease severity in patients with COVID-19. *Cytokine.* (2021) 137:155323. 10.1016/j.cyto.2020.155323 PMC7524708 33045526

[83] Sacchi A Grassi G Bordoni V Lorenzini P Cimini E Casetti R Early expansion of myeloid-derived suppressor cells inhibits SARS-CoV-2 specific T-cell response and may predict fatal COVID-19 outcome. *Cell Death Dis.* (2020) 11:921. 10.1038/s41419-020-03125-1 33110074 PMC7590570

[84] WHO Rapid Evidence Appraisal for COVID-19 Therapies (REACT) Working Group, Shankar-Hari M Vale C Godolphin P Fisher D Higgins J Association between administration of IL-6 antagonists and mortality among patients hospitalized for COVID-19: a meta-analysis. *JAMA.* (2021) 326:499–518. 34228774 10.1001/jama.2021.11330 PMC8261689

[85] Zhang X Tan Y Ling Y Lu G Liu F Yi Z Viral and host factors related to the clinical outcome of COVID-19. *Nature.* (2020) 583:437–40. 10.1038/s41586-020-2355-0 32434211

[86] De Biasi S Meschiari M Gibellini L Bellinazzi C Borella R Fidanza L Marked T cell activation, senescence, exhaustion and skewing towards TH17 in patients with COVID-19 pneumonia. *Nat Commun.* (2020) 11:3434. 10.1038/s41467-020-17292-4 32632085 PMC7338513

[87] Shimizu C Jain S Davila S Hibberd M Lin K Molkara D Transforming growth factor-beta signaling pathway in patients with Kawasaki disease. *Circ Cardiovasc Genet.* (2011) 4:16–25. 21127203 10.1161/CIRCGENETICS.110.940858 PMC3073847

[88] Dionne A Son M Randolph A. An update on multisystem inflammatory syndrome in children related to SARS-CoV-2. *Pediatr Infect Dis J.* (2022) 41:e6–9. 34889873 10.1097/INF.0000000000003393 PMC8658063

[89] Yang Y Peng F Wang R Guan K Jiang T Xu G The deadly coronaviruses: the 2003 SARS pandemic and the 2020 novel coronavirus epidemic in China. *J Autoimmun.* (2020) 109:102434. 10.1016/j.jaut.2020.102434 PMC7126544 32143990

[90] Moores L Tritschler T Brosnahan S Carrier M Collen J Doerschug K Prevention, diagnosis, and treatment of VTE in patients with coronavirus disease 2019: CHEST guideline and expert panel report. *Chest.* (2020) 158:1143–63. 10.1016/j.chest.2020.05.559 32502594 PMC7265858

[91] Carsana L Sonzogni A Nasr A Rossi R Pellegrinelli A Zerbi P Pulmonary post-mortem findings in a series of COVID-19 cases from northern Italy: a two-centre descriptive study. *Lancet Infect Dis.* (2020) 20:1135–40. 10.1016/S1473-3099(20)30434-5 32526193 PMC7279758

[92] ATTACC Investigators, ACTIV-4A Investigators, REMAP-CAP Investigators, Lawler P Goligher E Berger J Therapeutic anticoagulation with heparin in noncritically Ill patients with Covid-19. *N Engl J Med.* (2021) 385:790–802. 34351721 10.1056/NEJMoa2105911 PMC8362594

[93] Magro C Mulvey J Kubiak J Mikhail S Suster D Crowson A Severe COVID-19: a multifaceted viral vasculopathy syndrome. *Ann Diagn Pathol.* (2021) 50:151645. 10.1016/j.anndiagpath.2020.151645 PMC7553104 33248385

[94] Polak S Van Gool I Cohen D von der Thusen J van Paassen J. A systematic review of pathological findings in COVID-19: a pathophysiological timeline and possible mechanisms of disease progression. *Mod Pathol.* (2020) 33:2128–38. 32572155 10.1038/s41379-020-0603-3 PMC7306927

[95] D’Agnillo F Walters K Xiao Y Sheng Z Scherler K Park J Lung epithelial and endothelial damage, loss of tissue repair, inhibition of fibrinolysis, and cellular senescence in fatal COVID-19. *Sci Transl Med.* (2021) 13:eabj7790. 10.1126/scitranslmed.abj7790 34648357 PMC11000440

[96] Vaz de Paula C Nagashima S Liberalesso V Collete M da Silva F Oricil A COVID-19: immunohistochemical analysis of TGF-beta signaling pathways in pulmonary fibrosis. *Int J Mol Sci.* (2021) 23:168. 10.3390/ijms23010168 PMC8745764 35008594

[97] Colarusso C Maglio A Terlizzi M Vitale C Molino A Pinto A Post-COVID-19 patients who develop lung fibrotic-like changes have lower circulating levels of IFN-beta but higher levels of IL-1alpha and TGF-beta. *Biomedicines.* (2021) 9:1931. 10.3390/biomedicines9121931 34944747 PMC8698335

[98] Colarusso C Terlizzi M Maglio A Molino A Candia C Vitale C Activation of the AIM2 receptor in circulating cells of post-COVID-19 patients with signs of lung fibrosis is associated with the release of IL-1alpha, IFN-alpha and TGF-beta. *Front Immunol.* (2022) 13:934264. 10.3389/fimmu.2022.934264 35844548 PMC9277546

[99] Kvedaraite E Hertwig L Sinha I Ponzetta A Hed Myrberg I Lourda M Major alterations in the mononuclear phagocyte landscape associated with COVID-19 severity. *Proc Natl Acad Sci U.S.A.* (2021) 118:e2018587118. 10.1073/pnas.2018587118 33479167 PMC8017719

[100] Zhang X Topley N Ito T Phillips A. Interleukin-6 regulation of transforming growth factor (TGF)-beta receptor compartmentalization and turnover enhances TGF-beta1 signaling. *J Biol Chem.* (2005) 280:12239–45. 10.1074/jbc.M413284200 15661740

[101] Fabre T Kared H Friedman S Shoukry N. IL-17A enhances the expression of profibrotic genes through upregulation of the TGF-beta receptor on hepatic stellate cells in a JNK-dependent manner. *J Immunol.* (2014) 193:3925–33. 10.4049/jimmunol.1400861 25210118 PMC4185218

[102] Cucoranu I Clempus R Dikalova A Phelan P Ariyan S Dikalov S NAD(P)H oxidase 4 mediates transforming growth factor-beta1-induced differentiation of cardiac fibroblasts into myofibroblasts. *Circ Res.* (2005) 97:900–7. 10.1161/01.RES.0000187457.24338.3D 16179589

[103] Group R Horby P Lim W Emberson J Mafham M Bell J Dexamethasone in hospitalized patients with covid-19. *N Engl J Med.* (2021) 384:693–704. 32678530 10.1056/NEJMoa2021436 PMC7383595

[104] Wen F Kohyama T Skold C Zhu Y Liu X Romberger D Glucocorticoids modulate TGF-beta production. *Inflammation.* (2002) 26:279–90. 12546137 10.1023/a:1021412601538

[105] Ronit A Berg R Bay J Haugaard A Ahlstrom M Burgdorf K Compartmental immunophenotyping in COVID-19 ARDS: a case series. *J Allergy Clin Immunol.* (2021) 147:81–91. 10.1016/j.jaci.2020.09.009 32979342 PMC7581505

[106] Xiong Y Liu Y Cao L Wang D Guo M Jiang A Transcriptomic characteristics of bronchoalveolar lavage fluid and peripheral blood mononuclear cells in COVID-19 patients. *Emerg Microbes Infect.* (2020) 9:761–70. 32228226 10.1080/22221751.2020.1747363 PMC7170362

[107] Maloney J Stearman R Bull T Calabrese D Tripp-Addison M Wick M Loss-of-function thrombospondin-1 mutations in familial pulmonary hypertension. *Am J Physiol Lung Cell Mol Physiol.* (2012) 302:L541–54. 10.1152/ajplung.00282.2011 22198906 PMC3311532

[108] Carvacho I Piesche M. RGD-binding integrins and TGF-beta in SARS-CoV-2 infections - novel targets to treat COVID-19 patients? *Clin Transl Immunol.* (2021) 10:e1240. 10.1002/cti2.1240 33747508 PMC7971943

[109] Dinnon K III Leist S Okuda K Dang H Fritch E Gully K SARS-CoV-2 infection produces chronic pulmonary epithelial and immune cell dysfunction with fibrosis in mice. *Sci Transl Med.* (2022) 14:eabo5070. 10.1126/scitranslmed.abo5070 35857635 PMC9273046

[110] Gawaz A Guenova E. Microvascular skin manifestations caused by COVID-19. *Hamostaseologie.* (2021) 41:387–96. 34695855 10.1055/a-1581-6899

[111] Laurence J Nuovo G Racine-Brzostek S Seshadri M Elhadad S Crowson A Premortem skin biopsy assessing microthrombi, interferon type I antiviral and regulatory proteins, and complement deposition correlates with coronavirus disease 2019 clinical stage. *Am J Pathol.* (2022) 192:1282–94. 10.1016/j.ajpath.2022.05.006 35640675 PMC9144849

[112] Frumholtz L Bouaziz J Battistella M Hadjadj J Chocron R Bengoufa D Type I interferon response and vascular alteration in chilblain-like lesions during the COVID-19 outbreak. *Br J Dermatol.* (2021) 185:1176–85. 10.1111/bjd.20707 34611893 PMC8652826

[113] Wilkinson D Tsai W Schumacher M Barton M. Chromatin-bound p53 anchors activated Smads and the mSin3A corepressor to confer transforming-growth-factor-beta-mediated transcription repression. *Mol Cell Biol.* (2008) 28:1988–98. 10.1128/MCB.01442-07 18212064 PMC2268392

[114] Varga Z Flammer A Steiger P Haberecker M Andermatt R Zinkernagel A Endothelial cell infection and endotheliitis in COVID-19. *Lancet.* (2020) 395:1417–8. 32325026 10.1016/S0140-6736(20)30937-5 PMC7172722

[115] Rice L Padilla C McLaughlin S Mathes A Ziemek J Goummih S Fresolimumab treatment decreases biomarkers and improves clinical symptoms in systemic sclerosis patients. *J Clin Invest.* (2015) 125:2795–807. 10.1172/JCI77958 26098215 PMC4563675

[116] Chen W. A potential treatment of COVID-19 with TGF-beta blockade. *Int J Biol Sci.* (2020) 16:1954–5. 10.7150/ijbs.46891 32398962 PMC7211163

[117] Uckun F Saund S Windlass H Trieu V. Repurposing anti-malaria phytomedicine artemisinin as a COVID-19 drug. *Front Pharmacol.* (2021) 12:649532. 10.3389/fphar.2021.649532 33815126 PMC8017220

[118] Kim B Malek E Choi S Ignatz-Hoover J Driscoll J. Novel therapies emerging in oncology to target the TGF-beta pathway. *J Hematol Oncol.* (2021) 14:55. 10.1186/s13045-021-01053-x PMC8022551 33823905

[119] Dinnon K III Leist S Schafer A Edwards C Martinez D Montgomery S A mouse-adapted model of SARS-CoV-2 to test COVID-19 countermeasures. *Nature.* (2020) 586:560–6. 32854108 10.1038/s41586-020-2708-8 PMC8034761

[120] Imai M Iwatsuki-Horimoto K Hatta M Loeber S Halfmann P Nakajima N Syrian hamsters as a small animal model for SARS-CoV-2 infection and countermeasure development. *Proc Natl Acad Sci U.S.A.* (2020) 117:16587–95. 32571934 10.1073/pnas.2009799117 PMC7368255

[121] Munoz-Fontela C Dowling W Funnell S Gsell P Riveros-Balta A Albrecht R Animal models for COVID-19. *Nature.* (2020) 586:509–15. 32967005 10.1038/s41586-020-2787-6 PMC8136862

[122] Winkler E Bailey A Kafai N Nair S McCune B Yu J SARS-CoV-2 infection of human ACE2-transgenic mice causes severe lung inflammation and impaired function. *Nat Immunol.* (2020) 21:1327–35. 32839612 10.1038/s41590-020-0778-2 PMC7578095

[123] Morris J Tan A Olencki T Shapiro G Dezube B Reiss M Phase I study of GC1008 (fresolimumab): a human anti-transforming growth factor-beta (TGFbeta) monoclonal antibody in patients with advanced malignant melanoma or renal cell carcinoma. *PLoS One.* (2014) 9:e90353. 10.1371/journal.pone.0090353 24618589 PMC3949712

[124] Voelker J Berg P Sheetz M Duffin K Shen T Moser B Anti-TGF-beta1 antibody therapy in patients with diabetic nephropathy. *J Am Soc Nephrol.* (2017) 28:953–62. 10.1681/ASN.2015111230 27647855 PMC5328150

[125] Maden C Fairman D Chalker M Costa M Fahy W Garman N Safety, tolerability and pharmacokinetics of GSK3008348, a novel integrin alphavbeta6 inhibitor, in healthy participants. *Eur J Clin Pharmacol.* (2018) 74:701–9. 10.1007/s00228-018-2435-3 29532104 PMC5942357

[126] Kelley R Gane E Assenat E Siebler J Galle P Merle P A phase 2 study of galunisertib (TGF-beta1 receptor type I inhibitor) and sorafenib in patients with advanced hepatocellular carcinoma. *Clin Transl Gastroenterol.* (2019) 10:e00056. 10.14309/ctg.0000000000000056 31295152 PMC6708671

[127] Farah M Nelson K Tetzlaff M Nagarajan P Torres-Cabala C Prieto V Lichen planus related to transforming growth factor beta inhibitor in a patient with metastatic chondrosarcoma: a case report. *J Cutan Pathol.* (2020) 47:490–3. 10.1111/cup.13645 31930527

[128] Lacouture M Morris J Lawrence D Tan A Olencki T Shapiro G Cutaneous keratoacanthomas/squamous cell carcinomas associated with neutralization of transforming growth factor beta by the monoclonal antibody fresolimumab (GC1008). *Cancer Immunol Immunother.* (2015) 64:437–46. 10.1007/s00262-015-1653-0 25579378 PMC6730642

[129] Cappellini M Taher A. The use of luspatercept for thalassemia in adults. *Blood Adv.* (2021) 5:326–33. 33570654 10.1182/bloodadvances.2020002725 PMC7805339

[130] Vincenti F Fervenza F Campbell K Diaz M Gesualdo L Nelson P A Phase 2, double-blind, placebo-controlled, randomized study of fresolimumab in patients with steroid-resistant primary focal segmental glomerulosclerosis. *Kidney Int Rep.* (2017) 2:800–10. 10.1016/j.ekir.2017.03.011 29270487 PMC5733825

[131] Strauss J Gatti-Mays M Cho B Hill A Salas S McClay E Bintrafusp alfa, a bifunctional fusion protein targeting TGF-beta and PD-L1, in patients with human papillomavirus-associated malignancies. *J Immunother Cancer.* (2020) 8:e001395. 10.1136/jitc-2020-001395 33323462 PMC7745517

[132] Suto M Gupta V Mathew B Zhang W Pallero M Murphy-Ullrich J. Identification of Inhibitors of thrombospondin 1 activation of TGF-beta. *ACS Med Chem Lett.* (2020) 11:1130–6. 32550992 10.1021/acsmedchemlett.9b00540 PMC7294719

[133] Welsh B Faucette R Bilic S Martin C Schurpf T Chen D Nonclinical development of SRK-181: an anti-latent TGFbeta1 monoclonal antibody for the treatment of locally advanced or metastatic solid tumors. *Int J Toxicol.* (2021) 40:226–41. 33739172 10.1177/1091581821998945 PMC8135237

[134] Diop-Frimpong B Chauhan V Krane S Boucher Y Jain R. Losartan inhibits collagen I synthesis and improves the distribution and efficacy of nanotherapeutics in tumors. *Proc Natl Acad Sci U.S.A.* (2011) 108:2909–14. 10.1073/pnas.1018892108 21282607 PMC3041115

[135] van Andel M Indrakusuma R Jalalzadeh H Balm R Timmermans J Scholte A Long-term clinical outcomes of losartan in patients with Marfan syndrome: follow-up of the multicentre randomized controlled COMPARE trial. *Eur Heart J.* (2020) 41:4181–7. 10.1093/eurheartj/ehaa377 32548624 PMC7711887

[136] Puskarich M Ingraham N Merck L Driver B Wacker D Black L Efficacy of losartan in hospitalized patients with COVID-19-induced lung injury: a randomized clinical trial. *JAMA Netw Open.* (2022) 5:e222735. 10.1001/jamanetworkopen.2022.2735 35294537 PMC8928006

[137] Jaschinski F Rothhammer T Jachimczak P Seitz C Schneider A Schlingensiepen K. The antisense oligonucleotide trabedersen (AP 12009) for the targeted inhibition of TGF-beta2. *Curr Pharm Biotechnol.* (2011) 12:2203–13. 21619536 10.2174/138920111798808266

[138] Hamidi S Kadamboor Veethil S Hamidi S. Role of pirfenidone in TGF-beta pathways and other inflammatory pathways in acute respiratory syndrome coronavirus 2 (SARS-Cov-2) infection: a theoretical perspective. *Pharmacol Rep.* (2021) 73:712–27. 10.1007/s43440-021-00255-x 33880743 PMC8057922

[139] Zhang F Wei Y He L Zhang H Hu Q Yue H A trial of pirfenidone in hospitalized adult patients with severe coronavirus disease 2019. *Chin Med J (Engl).* (2021) 135:368–70. 10.1097/CM9.0000000000001614 34855641 PMC8812696

[140] Bogdahn U Hau P Stockhammer G Venkataramana N Mahapatra A Suri A Targeted therapy for high-grade glioma with the TGF-beta2 inhibitor trabedersen: results of a randomized and controlled phase IIb study. *Neuro Oncol.* (2011) 13:132–42. 10.1093/neuonc/noq142 20980335 PMC3018908

[141] Tian R Li Y Wang X Li J Li Y Bei S A Pharmacoinformatics analysis of artemisinin targets and de novo design of hits for treating ulcerative colitis. *Front Pharmacol.* (2022) 13:843043. 10.3389/fphar.2022.843043 35370688 PMC8971781

[142] Sadeghi A Tahmasebi S Mahmood A Kuznetsova M Valizadeh H Taghizadieh A Th17 and Treg cells function in SARS-CoV2 patients compared with healthy controls. *J Cell Physiol.* (2021) 236:2829–39. 32926425 10.1002/jcp.30047

[143] George P Wells A Jenkins R. Pulmonary fibrosis and COVID-19: the potential role for antifibrotic therapy. *Lancet Respir Med.* (2020) 8:807–15. 32422178 10.1016/S2213-2600(20)30225-3 PMC7228727

[144] Tolcher A Berlin J Cosaert J Kauh J Chan E Piha-Paul S A phase 1 study of anti-TGFbeta receptor type-II monoclonal antibody LY3022859 in patients with advanced solid tumors. *Cancer Chemother Pharmacol.* (2017) 79:673–80. 10.1007/s00280-017-3245-5 28280971 PMC5893148

[145] Crook H Raza S Nowell J Young M Edison P. Long covid-mechanisms, risk factors, and management. *BMJ.* (2021) 374:n1648. 10.1136/bmj.n1648 34312178

